# Variation in home range size of red foxes *Vulpes vulpes* along a gradient of productivity and human landscape alteration

**DOI:** 10.1371/journal.pone.0175291

**Published:** 2017-04-06

**Authors:** Zea Walton, Gustaf Samelius, Morten Odden, Tomas Willebrand

**Affiliations:** 1 Department of Forestry and Wildlife Management, Faculty of Applied Ecology and Agricultural Sciences, Inland Norway University of Applied Sciences, Campus Evenstad, Koppang, Norway; 2 Snow Leopard Trust, Seattle, Washington, United States of America; University of Sydney, AUSTRALIA

## Abstract

Home range size is a fundamental concept for understanding animal dispersion and ecological needs, and it is one of the most commonly reported ecological attributes of free-ranging mammals. Previous studies indicate that red foxes *Vulpes vulpes* display great variability in home range size. Yet, there has been little consensus regarding the reasons why home range sizes of red foxes vary so extensively. In this study, we examine possible causes of variation in red fox home range sizes using data from 52 GPS collared red foxes from four study areas representing a gradient of landscape productivity and human landscape alteration in Norway and Sweden. Using 90% Local Convex Hull home range estimates, we examined how red fox home range size varied in relation to latitude, elevation, vegetation zone, proportion of agricultural land and human settlement within a home range, and sex and age. We found considerable variation in red fox home range sizes, ranging between 0.95 km^2^ to 44 km^2^ (LoCoH 90%) and 2.4 km^2^ to 358 km^2^ (MCP 100%). Elevation, proportion of agricultural land and sex accounted for 50% of the variation in home range size found amongst foxes, with elevation having the strongest effect. Red foxes residing in more productive landscapes (those in more southern vegetation zones), had home ranges approximately four times smaller than the home ranges of foxes in the northern boreal vegetation zone. Our results indicate that home range size was influenced by a productivity gradient at both the landscape (latitude) and the local (elevation) scale. The influence of the proportion of agriculture land on home range size of foxes illustrates how human landscape alteration can affect the space use and distribution of red foxes. Further, the variation in home range size found in this study demonstrates the plasticity of red foxes to respond to changing human landscape alteration as well as changes in landscape productivity, which may be contributing to red fox population increases and northern range expansions.

## Introduction

The location and size of the area that an animal uses to secure resources and mates (i.e. its’ home range [1]) is fundamental to understanding animal dispersion and ecological needs. Home range size and location is also an important characteristic which structures species interactions, trophic processes and communities [2]. As such, home range size is one of the most commonly reported ecological attributes of free-ranging mammals [3].

Home range size can vary greatly across taxa, populations and individuals [4–7], and it is influenced by a complex array of ecological and social factors. Among carnivores, home range sizes have been found to vary by several orders of magnitude both within and among species [5, 8, 9]. Some of this variation has been attributed to differences in body mass [3, 10], population density [8, 11], prey availability [12, 13], environmental productivity and seasonality [14, 15], and intrinsic factors such as sex [16], reproductive status [17], and territoriality and social structure [18, 19].

However, there has been much debate as to the relative importance of these factors in shaping home range size, and often, such mechanisms are examined separately despite their synergistic effects on home range size [16, 20]. Thus, the factors influencing variation in home range size are still not well understood, especially across different scales [4, 21–23].

The red fox *Vulpes vulpes* is a species that demonstrates great flexibility in distribution, foraging behaviour and social structure [24–26]. Red foxes are highly adaptable habitat generalists with a distribution encompassing the entire northern hemisphere from arctic to temperate climes, and landscapes ranging from natural to exceedingly urban [27, 28]. Similarly, red foxes demonstrate a wide foraging niche as an opportunistic generalist predator. Further, they exhibit changing degrees of territorial behavior [25, 29] and display a complexity in their social structure ranging from pair bonding to family groups with helpers [26, 30].

Previous studies indicate that red foxes display high variability in home range size (see reviews in [26, 30]). Yet, there has been surprisingly little consensus as to the reasons why home range sizes of red foxes vary so extensively. Further, few studies have examined how the size of red fox home ranges may be influenced by changes along a landscape gradient (but see [8]). Landscape changes resulting from human alteration (e.g. agriculture, urbanization) and environmental productivity (increasing seasonality) have the ability to alter resource distribution as well as the availability and predictability of resources [8, 31, 32]. Furthermore, reductions in the availability of necessary resources can influence population density [15] and territoriality [4], which may alter social regulation and spacing patterns, thus leading to variation in home range size [4, 5]. Intersexual differences in response to spatial and temporal changes in resource distribution across landscapes can affect both individual and population demography [20, 33] and life history characteristics for a given species [34]. Thus, it is increasingly important to have a better understanding of sources of variation in red fox home range size along a productivity gradient.

Additionally, much of our previous knowledge regarding red fox spatial ecology has relied on VHF technology, with the choice of home range estimation technique and sampling scheme further influencing reported home range sizes [35, 36]. Advances in technology and analytical methods now allow for a more representative sample of an animal’s space use [36].

The objective of this study was to examine possible causes of variation in home range sizes of red foxes using data from 52 GPS collared foxes from four study areas representing a gradient of environmental productivity and human landscape alteration. Specifically, we examined variation in home range size in relation to extrinsic factors (latitude, elevation, vegetation zone, proportion of agricultural land and proportion of human settlement within a home range) and intrinsic factors (sex and age) of red foxes.

We predicted variation in home range sizes of red foxes along both a landscape (latitude) and localized (elevation) gradient, with home ranges being smallest in the south and increasing in size to the north and towards higher elevations, as increasing latitude and elevation have been shown to constrain environmental productivity and increase seasonality which alters resource availability [14, 33, 37]. Further, in more productive agricultural landscapes and areas of human settlement, resource needs can often be met within a smaller area [38, 39], thus, home range sizes were predicted to decrease with increasing proportion of agriculture land and human settlement.

## Methods

### Study areas

**Fig 1:**
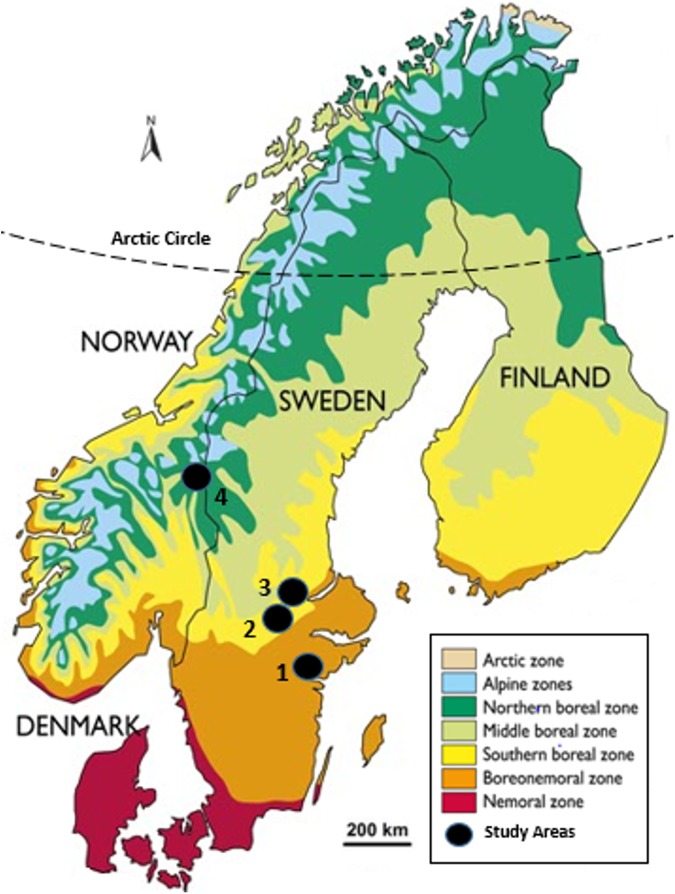
Study areas in Sweden and Norway classified by vegetation zone. The four study areas in Sweden and Norway along a landscape gradient from south to north classified within three vegetation zones: (1) Kolmården, (2) Grimsö, (3) Hedemora, and (4) Hedmark. Vegetation zone classifications were adapted from Moen and Lilletun [40] and Rydin et al. [41] for Norway and Sweden, respectively. Map is reprinted with permission from Hagen et al. [42].

We conducted this study within four different areas in Sweden and Norway representing a gradient of decreasing landscape productivity and human land use from Kolmården, Sweden, in the south to Hedmark County, Norway, in the north (58°–62° N; Fig 1). In general, the southernmost landscapes are more fragmented, consisting of boreonemoral forests, agricultural lands, and scattered human settlements, while the northern landscapes are characterized by boreal forests and alpine tundra of low diversity and productivity. Norway spruce (*Picea abies*) and Scots pine (*Pinus sylvestris*) dominate the forests in all areas, but birch (*Betula pubescens* and *B*. *verrucosa*) and other deciduous tree species are present increasingly to the south.

**Kolmården (58°40′N-16°22′E)** lies at an average altitude of 24 meters above sea level (a.s.l.), and the southern portion of the study area is coastal. This area is a mosaic of productive agriculture areas, boreonemoral forests and human settlements, thus representing a productive and more anthropogenically modified landscape. Daily mean temperatures range from 20°C in summer to –5°C in winter. Snow covers the ground irregularly from December to March.

**Grimsö (59°40'N-15°25'E)** and **Hedemora (60°16′N-15°59′E)** are both located in south-central Sweden and consist of a transitional border zone between boreonemoral forests in the south and boreal forests in the north. Grimsö is a 140km^2^ wildlife research area dominated by mixed coniferous forest (74%) and bogs (18%) with farmland comprising approximately 3% [43, 44]. Hedemora is located along the river Dalälven and contains settlements and productive agricultural areas along the river valley. Within both areas, the landscape is generally flat with altitude rising from 75m a.s.l. in the south to 180m a.s.l. in the north. Daily mean temperatures average 15°C in summer to –5°C in winter. The ground is generally snow covered from late December up to March.

**Hedmark County, Norway (61°53′N 12°2′E)** is a transitional border zone between northern boreal forest of low productivity and alpine tundra. The study area lies in the eastern part of Hedmark county, Norway, which extends from the Swedish border in the east to the Glomma River in the west. Most of the area lies 600m-800m a.s.l. Less than 1% of the area is cultivated or residential land, one third is productive forest, and the remainder consists of tundra, mountains, lakes, and rivers. Daily mean temperatures range from 10°C in summer to –25°C in winter, and the ground is generally snow covered from November to May.

### Fox capture

Between 2012 and 2016, we captured and equipped 80 red foxes with GPS radio collars (Tellus Ultralight, 210g, Televilt, Inc. Lindesberg, Sweden). Animal capture and handling protocols differed in Norway and Sweden, however all capture and handling procedures were approved by and followed the ethical guidelines required by the Swedish Animal Ethics Committee (permit numbers DNR 70–12, DNR 58–15) and the Norwegian Experimental Animal Ethics Committee (permit numbers 2009/122825, 2012/20038, 2014/207803). In addition, permits to capture wild animals were provided by the Norwegian Directorate for Nature Management and the Swedish Environmental Protection Board (NV-03459-11). All foxes were initially captured using baited wooden box traps. Foxes captured in Sweden were either immobilized using a mixture of 2 mg/kg ketamine and 0.08mg/kg medetomedine, where the medetomedine was later reversed with 0.4mg/kg atipamizole, or with 10 mg/kg tiletamine-zolazepam, for which there is no reversal [45]. In Norway, a noose pole was used to restrain captured foxes, which were then processed quickly and safely without chemical immobilization. Both capture methods were continuously refined to minimize handling time, animal stress and the risk of injury to the animals. Captured foxes were sexed, measured, weighed, and aged. Age was defined as sub-adult (< 1 year) or adult (> 1 year) based on the amount of tooth wear and tooth coloration. Only foxes meeting necessary weight requirements (>5kg) were fitted with radio collars. Total processing time of fox removal from the trap to fox release at capture site was approximately 25–35 minutes for Sweden and 10–15 minutes for Norway. Most foxes (88%) were captured between October and March. Collars deployed before October 2015 were programmed to take 3 positions per day with a drop-off after 270 days (9 months), and collars deployed after October 2015 were programmed to take 6 positions per day with a drop-off after 180 days (6 months). Four study animals were re-collared during the study period.

### Estimation of home range size

**Fig 2:**
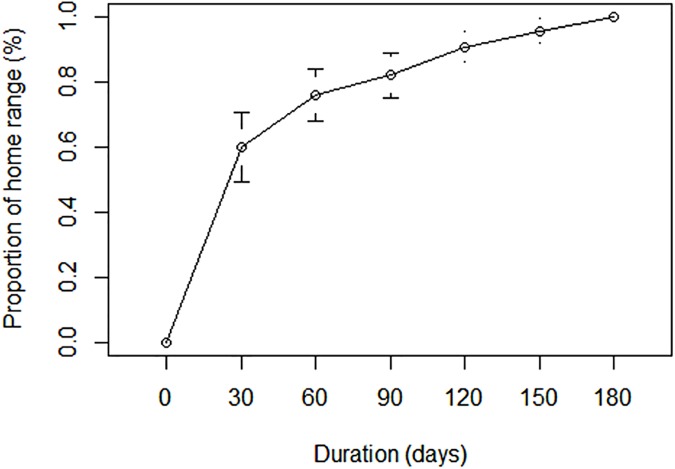
Area-observation curve showing variation in home range size in relation to sampling duration based on 100% MCP estimates of red foxes *Vulpes vulpes*. A duration of 90 days represented 82% of the home range size for red foxes followed for 6 months.

We determined the minimum monitoring duration of red foxes needed for home ranges to reach a stable asymptote based on area-observation curves [46]. This was done by using a subset of foxes monitored for >6 months (n = 15) to calculate when 100% Minimum Convex Polygon (MCP) estimates, using 30 day increments, started to reach an asymptote (Fig 2). Based on the area-observation curves, we restricted our analyses to foxes monitored ≥ 90 days (i.e. 3 months) that represented 82% of home range sizes for foxes monitored for 6 months. We did, however, include two females that were monitored for 84 and 87 days. Overall, mean monitoring duration of included foxes was 170 days ± 78 SD. We further limited our analyses to stationary foxes where we used a combination of visual inspection of the spatial data and net squared displacement (NSD) following Bunnefeld et al. [47] and Bastille-Rousseau et al [48] to identify different movement strategies corresponding to stationary, transient or dispersing foxes. In total, 52 foxes (M = 33, F = 19) met the requirements for inclusion in home range analysis.

We derived home ranges using two different non parametric methods: MCP, for comparison with previous red fox studies, and Local Convex Hull, a nonparametric kernel method using a fixed number of nearest neighboring points (LoCoH-k) [49, 50]. We chose to use LoCoH because it is more suitable for animals with home range borders that follow hard-edged features such as roads or rivers [50–52]. LoCoH estimates also exclude areas not likely to be utilized by an animal that may be included in MCP analysis [50]. For LoCoH-k estimates, individual k-values were calculated as the square root of the number of positions for each animal [49]. Using R 3.2.4 [53], we derived MCP and LoCoH-k home range estimates at 90%, 95% and 100% levels using the R package adehabitat [54].

### Home range composition

We obtained land cover and elevation from digitized topographic maps of Sweden (Swedish Land Cover, SMD, National Land Survey of Sweden) and Norway (Norwegian Mapping Authority). Using ArcGIS 10.1 [55], we converted the Swedish Land Cover map from raster data to vector layer by using the raster to polygon function. We then calculated the proportion of agricultural land and human settlement within each LoCoH-k 90% home range using the intersect function where surface areas were recalculated by using the calculate geometry function. Agriculture lands were defined as arable lands and pastures, and human settlements were localities of clustered inhabitants, buildings and associated infrastructure, forming small to large communities. Small amounts of arable lands within areas of human settlement, such as backyards, gardens, golf courses and parks were considered human settlements. Similarly, we calculated the mean elevation for home ranges by using (1) the zonal statistics as table function for home ranges in Sweden and (2) the intersect function of the elevation curves for home ranges in Norway, where the length of the elevation curves were recalculated using the calculate geometry function. We calculated the latitude for the centroid of each home range by using the polygon to point function. Finally, we used vegetation maps adapted from Moen and Lillethun [40] and Rydin et al. [41] for Norway and Sweden, respectively, to classify red fox home ranges according to the vegetation zone in which the home range was located. All fox home ranges were either located in the boreonemoral zone (BN), the southern boreal zone (SB), or the northern boreal zone (NB).

### Statistical analysis

We compared differences in mean home range sizes between all reciprocal levels of MCP and LoCoH-k estimates, and tested for statistical significance using a paired (Student’s) one-way t-test. For analysis of variation in home range size, we selected the more conservative 90% LoCoH home ranges estimates, (removing 10% of the outermost locations [56], as this level excluded extraterritorial movements which greatly expanded home range sizes at the 100% level (S1 Table, [49]). Further, Nilsen et al [57] cautioned against the use of MCP estimates for examining intraspecific sources of variation among home ranges. Therefore, by excluding occasional exploratory movements, the 90% LoCoH-k home range estimates probably produced more accurate depictions of the areas utilized by the animal than less conservative estimators [50]. We examined how home range size varied in relation to latitude and mean elevation, the proportion of agricultural land and the proportion of human settlement within a home range, and sex and age of foxes using these 90% LoCoH estimates and linear models in the program R (S2 Table [53]). Home range size was log transformed to achieve a more normal distribution of the data. We used a correlation matrix to evaluate collinearity among the fixed variables with a limit of (r ≥ 0.6). Latitude was highly correlated with elevation (Pearson’s r = 0.89). Elevation performed better than latitude when comparing full models (ΔAICc = 8.915), thus we retained elevation for further modeling. We derived 31 candidate models from the independent variables above, excluding latitude, and ranked the models based on the Akaike’s Information Criterion with small sample adjustment (AIC_C_) [58] using the R package MuMin [59]. We selected the model with the lowest AIC_C_ value as the best model though we considered models within two AIC_C_ units to be of similar quality [58]. Model assumptions were checked and final models were validated by examining the residuals.

## Results

### Home range size estimates

Red fox home ranges showed considerable variation in size between the different home range estimators and among individuals (S1 Table). MCP estimates were significantly larger than the corresponding LoCoH-k home ranges at all levels (paired *t-*test, 90% t_51_ = 3.13, p = 0.003, 95% t_51_ = 2.96, p = 0.005, 100% t_51_ = 3.35, p = 0.002). The GPS data emphasized the occurrence of excursions and exploratory movement patterns, which resulted in outlying positions greatly increasing home range sizes, depending on the estimator used. LoCoH-k estimates decrease substantially when outlying fixes were removed, compared to MCP estimates, resulting in overall more conservative home range size estimates. Specifically, using 90% of the core relocations resulted in the average MCP home range size almost triple the size compared to LoCoH-k estimates (13km^2^ difference; Table 1).

**Table 1:** Mean home range sizes of red foxes.

	Mean Home Range Size (km^2^)					
	Mean ± SE Range		Mean ± SE Range		Mean ± SE Range	
**LoCoH-k**	**90%**		**95%**		**100%**	
All Foxes (n = 52)	7.1 ± 1.3	(1.0–44)	11 ± 1.9	(1.3–63)	32 ± 5.4	(1.9–185)
Females (n = 19)	8.0 ± 2.6	(1.0–44)	11 ± 3.5	(1.3–57)	27 ± 10	(1.9–185)
Males (n = 33)	6.6 ± 1.3	(1.0–35)	11 ± 2.3	(1.9–63)	35 ± 6.2	(4.7–114)
**MCP**	**90%**		**95%**		**100%**	
All Foxes (n = 52)	20 ± 5.2	(1.5–193)	26 ± 6.7	(1.9–273)	52 ± 10	(2.4–358)
Females (n = 19)	16 ± 5.1	(1.7–77)	20 ± 6.7	(1.9–111)	33 ± 12	(2.4–206)
Males (n = 33)	23 ± 7.6	(1.5–193)	30 ± 9.8	(2.6–273)	63 ± 15	(6.0–358)

Mean home range sizes of red foxes *Vulpes vulpes* based on 90%, 95% and 100% Local Convex Hull (LoCoH-k) and Minimum Convex Polygon (MCP) estimates. Estimates are for all study areas combined. Standard error (SE) and range of minimum to maximum home range sizes are also provided.

Red fox home ranges in this study showed considerable individual variation in size as well, ranging between 0.95 km^2^ to 44 km^2^ (LoCoH 90%) and 2.4 km^2^ to 358 km^2^ (MCP 100%). Home ranges averaged 7.1 km^2^ ± 1.3 SE (90% LoCoH-k) or 52 km^2^ ± 10 SE (100% MCP) which varied depending on estimator and level (Table 1). In general, the home ranges of red foxes in more productive vegetation zones (i.e. those in the boreonemoral and the southern boreal vegetation zones) were approximately four times smaller than home ranges of foxes in the northern boreal zone (90% LoCoH-k), and this trend held independent of estimator or proportion of relocations included in the estimates (Table 2). Only three red fox home ranges located in the three southern study areas were larger than 10 km^2^ (90% LoCoH-k, n = 44) while only one home range in the northern study area was smaller than 10 km^2^ (90% LoCoH-k, range = 8.3–44 km^2^, n = 8).

**Table 2:** Mean home range size estimates (LoCoH-k 90%) of red foxes according to the different vegetation zones they occurred in, listed from south to north.

Vegetation Zone	Home range size (km^2^)	Human Settlement	Agriculture Land	Mean Elevation (m)
**Boreonemoral**				
All Foxes (n = 30)	**4.7 ± 4.7**	**1% ± 4%**	**28% ± 21%**	**54 ± 22**
Male (n = 21)	5.4 ± 5.8	2% ± 4%	26% ± 21%	54 ± 24
Female (n = 9)	3.0 ± 1.4	0% ± 0%	33% ± 22%	54 ± 19
**Southern Boreal**				
All Foxes (n = 14)	**5.2 ± 8.6**	**7% ± 15%**	**25% ± 21%**	**106 ± 40**
Male (n = 9)	6.5 ± 10.6	4% ± 9%	26% ± 22%	118 ± 42
Female (n = 5)	2.7 ± 2.0	11% ± 22%	22% ± 22%	84 ± 29
**Northern Boreal**				
All foxes (n = 8)	**19.5 ± 11.8**	**0% ± 0%**	**2% ± 2%**	**605 ± 164**
Male (n = 3)	14.7 ± 5.9	0% ± 0%	1% ± 1%	454 ± 195
Female (n = 5)	22.5 ± 14.0	0% ± 0%	3% ± 2%	695 ± 25

Mean LoCoH-k 90% home range size estimates of red foxes according to the different vegetation zones they occurred in. Also shown are the differences in mean proportion of human settlement and agriculture within home ranges (%) and mean elevation (m) for each vegetation zone. Vegetation zones are listed from south to north, with sample sizes (n) and standard deviations (SD) also provided.

### Home range variation along a gradient

The red fox home ranges in this study contained on average 23% agricultural land (± 0.21SD, range 0%-76%) and 3% human settlement (± 0.08SD, range 0%-50%), which varied along a landscape gradient (Table 2). Notably, only 11 of 52 foxes had home ranges containing >1% human settlement while more than a third of the foxes (n = 22 of 52) had less than 10% agriculture land within their home ranges. Eighty-five percent of the red fox home ranges were situated below 200m elevation, with the remaining 15% (n = 8 foxes) only occurring in the northern boreal vegetation zone at elevations above 200m (range 264m-729m).

### Variation in home range size

**Fig 3:**
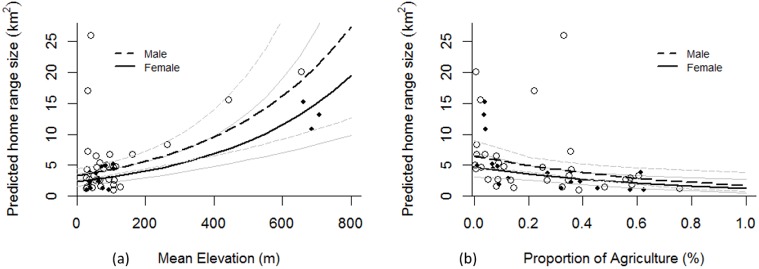
Predicted changes in red fox (*Vulpes vulpes*) home range size in relation to increasing elevation and proportion of agriculture land. Predicted changes in home range size (LoCoH-k 90%) of female and male red foxes in relation to increasing elevation (a) and proportion of agriculture (b). Female home range values are indicated by the solid black dots and male home ranges are open circles. Regression lines (dashed black for males and solid black for females) are from backtransformed model estimates with the 95% C.I.s of predicted values shown (solid or dashed gray lines, for females and males, respectively).

The best ranked model of home range size variation included mean elevation, proportion of agriculture land and sex, and accounted for 50% of the variation in home range size amongst foxes (R^2^ = 0.50) and 52% of cumulative model weight (Table 3). Mean elevation had the strongest effect on home range size with home ranges increasing by 0.3 km^2^ when elevation increased by 100 m (β_1_ = 0.003 ± 0.001 SE, Fig 3A). A 10% increase in the proportion of agriculture land within a home range resulted in a decrease of 0.14 km^2^ in home range size (β_2_ = -1.37 ± 0.51 SE, Fig 3B). The inclusion of sex improved the final model by 0.3 ΔAICc over the second ranked model, but there was little difference in home range size between sexes (β_3_ = 0.34 ± 0.21 SE, Fig 3).

**Table 3:** Model selection for variables affecting variation in home range size of red foxes.

Model	df	logLik	AICc	ΔAICc	AICω
Elevation + Agriculture + Sex	5	-52.28	115.87	0	0.28
Elevation + Agriculture	4	-53.67	116.20	0.33	0.24
Elevation + Agriculture + Human settlement	5	-53.30	117.91	2.05	0.10
Elevation + Agriculture + Human settlement + Sex	6	-52.03	117.93	2.06	0.10
Elevation + Agriculture + Human settlement + Sex + Age	6	-52.05	117.96	2.09	0.09
Elevation + Agriculture + Age	5	-53.52	118.34	2.47	0.08
(*Null*)	*2*	*-71*.*86*	*147*.*96*	*32*.*09*	*0*.*00*

Model selection for variables affecting variation in home range size of red foxes using LoCoH-k 90% home range estimates with the covariates: sex, age, proportion agriculture land within home range (agriculture), proportion human settlement within home range (human settlement) and mean elevation (elevation). Presented models had a delta AICc value <4, except the null model (in italics) which is provided for comparision. Model selection was based on ΔAICc values and model weights (ω) with models ≤ 2 ΔAICc considered equivalent.

## Discussion

The red fox home range sizes in our study were much larger than those previously reported for red foxes [26, 30]. Remarkably, our average 100% MCP home range size estimate for all foxes (52 km^2^) was three times larger than estimates from comparable studies of red foxes, in similar boreal and tundra landscapes [60–62]. Our use of GPS technology may have detected excursions and outlying positions to a greater degree than previous studies using VHF technology, which may have limited previous estimates of home range size. LoCoH-k estimates decreased substantially when outlying fixes were removed, compared to MCP estimates, and our average 90% LoCoH estimates were of similar size to 100% MCP estimates from the comparable studies above.

Furthermore, the GPS collars used in this study are not limited to our ability to follow and relocate animals. They therefore have the ability to increase our knowledge of movements and behaviors across landscapes and far distances. Our use of GPS technology not only highlights larger sized home ranges than previously known, but also the exploratory movement patterns of red foxes, indicating that excursions may be more common among red foxes than previously thought. Several foxes within our study showed a pattern of utilizing multiple, separate core areas within large home ranges. Further, six foxes, not included in this study, used two distinct home ranges and regularly traveled between them. Meia and Weber [63] cautioned the use of nomadic foxes in averaging home range estimates due to the significant home range size differences between resident and nomadic foxes. However, this study shows movement patterns that indicate resident foxes use much larger areas than previously presumed. ‘Nomadic’ foxes may actually be resident foxes traveling between core areas of resources within very large home ranges. These spatial patterns demonstrate the ability of GPS collars to enhance our knowledge of red fox movements and behaviors across landscapes, and highlight the flexibility of red foxes in their space use. This further challenges the traditional home range concept for a highly adaptable, generalist predator such as the red fox and warrants further attention.

Red fox home ranges at higher elevations and in the northern boreal vegetation zone were approximately four times larger than those of foxes at lower elevations and in the two southern vegetation zones, indicating that home range size was influenced by a productivity gradient at both the landscape (latitude) and the local (elevation) scales. Larger home ranges at higher latitudes and elevations have also been found for wolves in Scandinavia [7], and this pattern has been found in ungulates as well [64].

Elevation showed the strongest effect on home range size. This is possibly because the changes in environmental productivity along a latitude gradient were not as evident as the environmental variation (snow cover and seasonality) experienced at a local scale with increasing elevation. Further, increasing seasonality has been found to decrease population density [15]. It may be that foxes with large home ranges in the high elevation, northern study area are not constrained in their space use by social regulating factors, mediated through population density or territoriality [65], which could further restrict space use patterns of foxes in the more southern and productive study areas.

At lower elevations, where the amount of available agricultural land increased, red foxes with a higher proportion of agriculture land maintained smaller home ranges. Fragmented agricultural landscapes often allow for higher prey densities compared to northern areas dominated by boreal forest [39] and increased habitat heterogeneity can allow for resource needs to be met within smaller areas [66]. Studies of other mid-sized canids have shown smaller home ranges near human settlements compared to natural areas due to increased resource availability [6, 67, 68].

Red foxes did not demonstrate clear intersexual differences in home range size. However, the inclusion of sex in the final model indicates intersexual differences within elevation gradients and proportion of agricultural land in home ranges. We do not know breeding status of female foxes, thus it is possible the impact of sex may have been related to seasonal differences related to breeding status [17] or an artifact of sample size as fewer female foxes were monitored. Similarly, there was a sex mismatch between home range estimators, where the maximum home range sizes using LoCoH-k estimates (90% and 100%) belonged to female foxes, and the upper values of MCP estimates at the same levels belonged to male foxes. This indicates that home range size was affected by sexual differences in movement patterns, or possible underlying behavioral differences, which could in turn lead to over/under-representation of home range size depending on the method of estimation.

The overall flexibility of the red fox in its space use, social structure and resource utilization makes disentangling the sources of intraspecific variation in home range size complex. Further, both population density [69] and territoriality [4] are key intrinsic factors that can decrease home range sizes. These may be altered by human influences and lethal control of populations [70]. Hunting pressure and human attitudes towards red foxes can further impact the relationship between resource availability and home range size [9]. Nevertheless, our study clearly demonstrates the importance of environmental productivity and seasonality to red fox space use. The pronounced variation in home range size illustrates the plasticity of red foxes’ space use, and this trait may enhance their ability to respond to both climate and human mediated landscape changes and facilitate red fox population increases and northern range expansions [71, 72]. While this study provides insight into possible mechanisms underlying variation in red fox home range size, the influence of a such a generalist species, and its’ potential for population expansion warrants further attention [73, 74].

## Supporting information

S1 Table Summary of home range sizes of all red foxes included in this study. Summary of home range sizes of all red foxes (*Vulpes vulpes*) included in this study. All foxes were resident individuals that were monitored ≥ 3 months, with the exception of two females monitored for 84 and 87 days (fox number 16 and 41). The LoCoH-k 90% estimates highlighted in grey represent the home range sizes used in data analysis and modeling.
(DOCX)

S2 Table Fox data and metadata. (XLSX)
