# Supplementary material for: Variation in home range size of red foxes Vulpes vulpes along a gradient of productivity and human landscape alteration
Source: PLoS One. 2017 Apr 6;12(4):e0175291. doi: 10.1371/journal.pone.0175291 (PMC5383297; doi:10.1371/journal.pone.0175291)
Supplement: S1 Table — Summary of home range sizes of all red foxes (Vulpes vulpes) included in this study. All foxes were resident individuals that were monitored ≥ 3 months, with the exception of two females monitored for 84 and 87 days (fox number 16 and 41). The LoCoH-k 90% estimates highlighted in grey represent the home range sizes used in data analysis and modeling. (DOCX) [file pone.0175291.s001.docx]

| **S1 Table. Summary of home range sizes of red foxes (*Vulpes vulpes*) included in this study.** All foxes were resident individuals that were monitored ≥ 3 months, with the exception of two females monitored for 84 and 87 days (fox number 16 and 41). The LoCoH-k 90% estimates highlighted in grey represent the home range sizes used in data analysis and modeling. | | | | | | | | | | | | | | | |  |
| --- | --- | --- | --- | --- | --- | --- | --- | --- | --- | --- | --- | --- | --- | --- | --- | --- |
|  | **Animal Details** | | | | | **Monitoring Details** | | | | **Home Range Estimates (km^2^)** | | | | | | |
| **Fox No.** | | **Name** | **Study Area** | **Sex** | **Age** | **No. Pos.** | **No. Days** | | **MCP 90%** | **MCP 95%** | **MCP 100%** | **LoCoH 90%** | **LoCoH 95%** | **LoCoH 100%** | **k** | |
| **1** | | Ann* | Kolmården | F | AD | 762 | 337 | 3.1 | | 4.0 | 9.4 | 2.2 | 2.7 | 4.7 | 28 | |
| **2** | | Bengt | Kolmården | M | AD | 254 | 91 | 12.4 | | 12.9 | 16.1 | 4.4 | 5.4 | 14.1 | 16 | |
| **3** | | Bosse | Kolmården | M | AD | 626 | 218 | 27.9 | | 48.3 | 212 | 7.2 | 23.0 | 105 | 25 | |
| **4** | | Cristina | Kolmården | F | SA | 764 | 324 | 5.6 | | 7.2 | 9.5 | 3.7 | 4.8 | 7.7 | 28 | |
| **5** | | Donald | Kolmården | M | AD | 657 | 114 | 193 | | 273 | 358 | 26.0 | 44.3 | 105 | 26 | |
| **6** | | Fantomen | Kolmården | M | AD | 1139 | 203 | 32.0 | | 32.0 | 201 | 6.5 | 10.3 | 89.0 | 34 | |
| **7** | | Frans | Kolmården | M | AD | 310 | 148 | 5.0 | | 8.0 | 14.0 | 4.7 | 6.2 | 12.5 | 18 | |
| **8** | | Fredrik | Kolmården | M | AD | 913 | 173 | 6.0 | | 6.0 | 10.0 | 3.3 | 4.2 | 9.0 | 30 | |
| **9** | | Gunilla* | Kolmården | F | AD | 379 | 163 | 8.6 | | 9.0 | 9.8 | 4.8 | 5.5 | 9.1 | 19 | |
| **10** | | Gustaf | Kolmården | M | AD | 581 | 230 | 7.4 | | 10.4 | 18.7 | 4.3 | 6.5 | 14.3 | 24 | |
| **11** | | Ingvar* | Kolmården | M | AD | 1093 | 479 | 8.5 | | 9.1 | 16.9 | 5.4 | 7.0 | 14.0 | 33 | |
| **12** | | Ivan | Kolmården | M | SA | 346 | 150 | 3.5 | | 7.4 | 20.6 | 2.6 | 4.8 | 15.1 | 19 | |
| **13** | | Janne | Kolmården | M | AD | 360 | 141 | 2.0 | | 2.6 | 8.2 | 1.3 | 1.9 | 7.8 | 19 | |
| **14** | | Kenneth | Kolmården | M | AD | 732 | 271 | 4.4 | | 4.9 | 6.0 | 3.0 | 3.8 | 5.6 | 27 | |
| **15** | | Kerstin | Kolmården | F | AD | 269 | 128 | 5.6 | | 5.8 | 6.2 | 2.8 | 3.4 | 5.6 | 16 | |
| **16** | | Klara | Kolmården | F | SA | 236 | 84 | 1.7 | | 2.2 | 2.6 | 1.3 | 1.5 | 2.1 | 15 | |
| **17** | | Klas | Kolmården | M | SA | 400 | 141 | 2.5 | | 5.9 | 9.1 | 1.2 | 1.9 | 4.8 | 20 | |
| **18** | | Kula | Kolmården | M | SA | 469 | 169 | 8.8 | | 18.5 | 114 | 6.8 | 14.9 | 70.1 | 22 | |
| **19** | | Lollo | Kolmården | F | SA | 371 | 146 | 4.0 | | 5.0 | 6.0 | 2.4 | 3.7 | 4.8 | 19 | |
| **20** | | Niklas* | Kolmården | M | SA | 729 | 273 | 8.9 | | 9.7 | 10.4 | 5.1 | 6.9 | 9.2 | 27 | |
| **21** | | Örjan | Kolmården | M | SA | 1049 | 181 | 73.0 | | 95.0 | 172 | 17.1 | 26.9 | 104 | 32 | |
| **22** | | Oskar | Kolmården | M | SA | 319 | 118 | 4.3 | | 5.7 | 57.4 | 2.6 | 4.1 | 42.6 | 18 | |
| **23** | | Ronja | Kolmården | F | AD | 584 | 271 | 10.5 | | 11.7 | 14.3 | 4.9 | 6.4 | 10.9 | 24 | |
| **24** | | Sam | Kolmården | M | AD | 251 | 102 | 3.7 | | 4.0 | 15.8 | 2.8 | 3.5 | 12.1 | 16 | |
| **25** | | Spank | Kolmården | M | AD | 506 | 192 | 6.9 | | 8.6 | 26.1 | 4.7 | 6.9 | 16.8 | 22 | |
| **Animal Details** | | | | | **Monitoring Details** | | | | | **Home Range Estimates (km^2^)** | | | | | | |
| **Fox No.** | | **Name** | **Study Area** | **Sex** | **Age** | **No. Pos.** | **No. Days** | **MCP 90%** | | **MCP 95%** | **MCP 100%** | **LoCoH 90%** | **LoCoH 95%** | **LoCoH 100%** | **k** | |
| **26** | | Tarzan | Kolmården | M | AD | 1024 | 184 | 4.0 | | 11.0 | 70.0 | 2.7 | 7.8 | 51.2 | 32 | |
| **27** | | Tessan | Kolmården | F | AD | 264 | 97 | 1.7 | | 1.9 | 2.4 | 1.0 | 1.3 | 1.9 | 16 | |
| **28** | | Theo | Kolmården | M | AD | 306 | 112 | 8.2 | | 8.3 | 16.8 | 1.5 | 3.0 | 12.3 | 17 | |
| **29** | | Viktor | Kolmården | M | AD | 455 | 164 | 3.1 | | 3.3 | 52.5 | 1.4 | 2.2 | 34.2 | 21 | |
| **30** | | Wilma | Kolmården | F | AD | 401 | 236 | 17.8 | | 20.9 | 29.8 | 3.8 | 5.8 | 15.9 | 20 | |
| **31** | | Carmen | Grimsö | F | SA | 671 | 277 | 8.5 | | 10.4 | 16.2 | 5.2 | 6.5 | 12.8 | 26 | |
| **32** | | Espen | Grimsö | M | SA | 423 | 200 | 6.1 | | 7.2 | 11.0 | 2.6 | 3.9 | 8.3 | 21 | |
| **33** | | Farbror | Grimsö | M | AD | 209 | 101 | 4.3 | | 5.2 | 12.2 | 2.7 | 3.9 | 8.5 | 14 | |
| **34** | | Gijom | Grimsö | M | SA | 491 | 174 | 5.5 | | 8.3 | 20.3 | 3.4 | 4.5 | 15.2 | 22 | |
| **35** | | Helge | Grimsö | M | SA | 704 | 129 | 12.0 | | 29.0 | 76.0 | 6.8 | 19.4 | 59.4 | 27 | |
| **36** | | Ida | Grimsö | F | AD | 833 | 191 | 7.0 | | 9.0 | 20.0 | 4.4 | 6.1 | 18.2 | 29 | |
| **37** | | Gunde | Hedemora | M | SA | 252 | 94 | 25.1 | | 27.2 | 36.1 | 15.6 | 18.9 | 26.9 | 16 | |
| **38** | | Jörgen | Hedemora | M | SA | 510 | 212 | 9.0 | | 11.1 | 24.9 | 4.8 | 6.4 | 18.2 | 23 | |
| **39** | | Kalla | Hedemora | F | SA | 289 | 96 | 1.9 | | 2.3 | 22.2 | 0.95 | 1.3 | 13.3 | 17 | |
| **40** | | Lycka | Hedemora | F | AD | 245 | 90 | 2.4 | | 2.9 | 3.8 | 1.2 | 2.1 | 3.2 | 16 | |
| **41** | | Magda | Hedemora | F | SA | 232 | 87 | 6.5 | | 9.0 | 24.0 | 1.8 | 2.8 | 11.9 | 15 | |
| **42** | | Masen | Hedemora | M | SA | 260 | 94 | 1.5 | | 3.1 | 18.6 | 0.97 | 2.5 | 11.6 | 16 | |
| **43** | | Nås | Hedemora | M | AD | 339 | 118 | 2.5 | | 3.1 | 6.0 | 1.4 | 2.0 | 4.7 | 18 | |
| **44** | | Pilkoms | Hedemora | M | AD | 583 | 101 | 3.0 | | 4.0 | 8.0 | 1.8 | 2.6 | 7.0 | 24 | |
| **45** | | Wilhelm | Hedemora | M | AD | 526 | 91 | 163 | | 174 | 220 | 34.5 | 62.7 | 114 | 23 | |
| **46** | | Bane | Hedmark | M | SA | 527 | 97 | 71.2 | | 92.1 | 145 | 20.1 | 29.3 | 92.9 | 23 | |
| **47** | | Engerdal | Hedmark | F | AD | 339 | 136 | 76.6 | | 111 | 206 | 44.0 | 56.9 | 185 | 18 | |
| **48** | | Snerta | Hedmark | F | SA | 600 | 268 | 30.2 | | 43.2 | 51.9 | 15.2 | 21.6 | 41.0 | 24 | |
| **49** | | Sømå | Hedmark | F | AD | 445 | 105 | 17.0 | | 20.0 | 34.0 | 10.8 | 14.1 | 33.6 | 21 | |
| **50** | | Svala | Hedmark | F | AD | 973 | 175 | 75.0 | | 83.0 | 122 | 29.1 | 45.2 | 106 | 31 | |
| **51** | | Tallåsa | Hedmark | F | AD | 962 | 184 | 18.3 | | 23.2 | 32.2 | 13.1 | 16.7 | 26.4 | 31 | |
| **52** | | Tufsing | Hedmark | M | SA | 478 | 186 | 21.6 | | 26.3 | 79.1 | 8.4 | 15.0 | 46.0 | 22 | |
| ***** Fox was recollared during the study period and data combined for analysis. | | | | | | | |  | |  |  |  |  |  |  | |
